# Five-year disease-modifying therapeutic experience of 102 Chinese paediatric 5q-spinal muscular atrophy: a retrospective analysis

**DOI:** 10.1093/braincomms/fcaf453

**Published:** 2025-11-27

**Authors:** Minyan Jiang, Cuili Liang, Yani Zhang, Kelu Zheng, Kaishou Xu, Lu He, Jianping Tao, Weizhe Wu, Ruidan Zheng, Min Rao, Wen Zhang, Wenhao Zhou, Li Liu

**Affiliations:** Department of Paediatric Endocrinology and Genetic Metabolism, Guangzhou Women and Children’s Medical Center, Guangzhou Medical University, Guangzhou 510623, China; Department of Paediatric Endocrinology and Genetic Metabolism, Guangzhou Women and Children’s Medical Center, Guangzhou Medical University, Guangzhou 510623, China; Department of Paediatric Neurology, Guangzhou Women and Children’s Medical Center, Guangzhou Medical University, Guangzhou 510623, China; Department of Paediatric Neurology, Guangzhou Women and Children’s Medical Center, Guangzhou Medical University, Guangzhou 510623, China; Department of Paediatric Physiotherapy, Guangzhou Women and Children’s Medical Center, Guangzhou Medical University, Guangzhou 510623, China; Department of Paediatric Physiotherapy, Guangzhou Women and Children’s Medical Center, Guangzhou Medical University, Guangzhou 510623, China; Department of Paediatric Intensive Care Unit, Guangzhou Women and Children’s Medical Center, Guangzhou Medical University, Guangzhou 510623, China; Department of Pharmacology, Guangzhou Women and Children’s Medical Center, Guangzhou Medical University, Guangzhou 510623, China; Department of Paediatric Endocrinology and Genetic Metabolism, Guangzhou Women and Children’s Medical Center, Guangzhou Medical University, Guangzhou 510623, China; Department of Paediatric Endocrinology and Genetic Metabolism, Guangzhou Women and Children’s Medical Center, Guangzhou Medical University, Guangzhou 510623, China; Department of Paediatric Endocrinology and Genetic Metabolism, Guangzhou Women and Children’s Medical Center, Guangzhou Medical University, Guangzhou 510623, China; Department of Neonatal, Guangzhou Women and Children’s Medical Center, Guangzhou Medical University, Guangzhou 510623, China; Department of Paediatric Endocrinology and Genetic Metabolism, Guangzhou Women and Children’s Medical Center, Guangzhou Medical University, Guangzhou 510623, China

**Keywords:** 5q-spinal muscular atrophy, SMN, nusinersen, Chinese

## Abstract

5q-spinal muscular atrophy (SMA) is a fatal autosomal recessive disease characterized by the progressive muscle weakness and atrophy. In this retrospective study, we described the long-term clinical outcomes of novel disease-modifying therapies (DMTs) for 5q-spinal muscular atrophy, drawing on experience from southern China. This is a single-centre large cohort which enrolled 102 paediatric patients confirmed with 5q-spinal muscular atrophy at Guangzhou Women and Children’s Medical Center from 2019 to 2024. One hundred and two patients were included, 24 were classified as SMA type 1, 56 with type 2 and 22 with type 3. One hundred per cent of the patients received nusinersen, with 31 (30.3%) patients starting risdiplam and 2 patients transitioning to zolgensma therapy. Over the 5-year treatment and follow-up period (2019–24), the survival rate reached 97.08%. One child with SMA type 1 and two with SMA type 2 died while receiving nusinersen monotherapy. Compared with baseline, the enrolled SMA patients exhibited statistically significant motor function gains. Nevertheless, type 1 patients experienced weight loss, while linear growth was compromised in both type 1 and type 2 patients after treatment. Serum insulin-like growth factor-1 levels rose modestly in types 1 and 2, but the increase did not reach statistical significance. Respiratory tract infections, malnutrition, scoliosis and fracture are the main complications and potential life-threatening risk factors during DMTs. Moreover, longer diagnostic to treatment intervals were significantly and inversely associated with motor function gains and directly associated with higher complication rates. This retrospective study confirms the effectiveness of nusinersen and risdiplam for 5q-spinal muscular atrophy and highlights the critical importance of early initiation of DMTs.

## Teaser

Jiang *et al*. showed that 5-year follow-up data from 102 children with 5q-spinal muscular atrophy who received disease-modifying therapy revealed improvements in motor function, growth status and frequency of complications, thereby underscoring the highly variable clinical outcomes observed in this patient cohort.

## Graphical Abstract

**Graphical Abstract:**
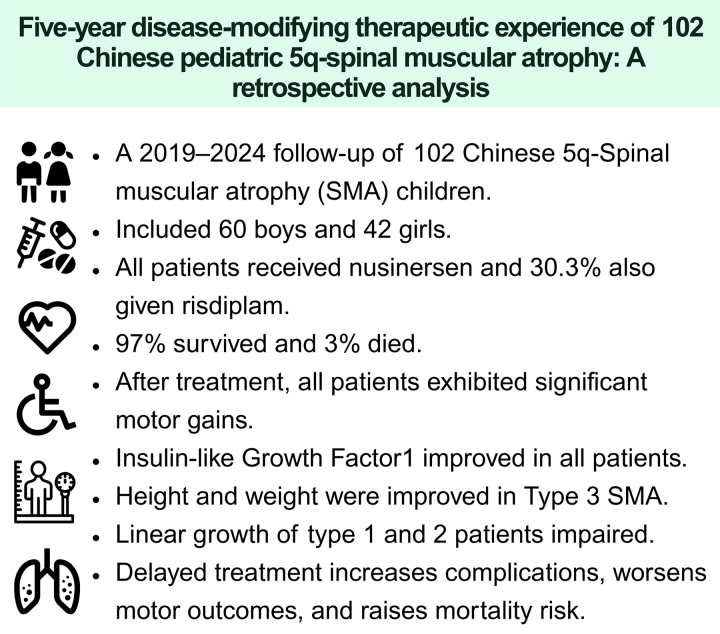

## Introduction

Spinal muscular atrophy (SMA) caused by a homozygous deletion or mutation in the survival motor neuron (SMN) 1 gene on chromosome 5q13.2 is the most common childhood-onset hereditary motor neuron disease.^1^ The disease is characterized by progressive muscle weakness and atrophy, including skeletal muscles of the limbs and trunk and of the bulbar and respiratory muscle due to the degeneration of anterior horn cells.^2^ There is no cure in the past decade.^3^

Currently, disease-modifying therapies (DMTs) are available for the treatments of 5q-SMA. In China, nusinersen, as the first pharmacologic treatment was approved in February 2019. Subsequently, risdiplam was marked in June 2021. Onasemnogene abeparvovec as gene therapy approved by the US FDA in 2019 is still in the clinical trial stage in China.

Owing to differences in DMT policies and the natural disease course of SMA among different populations, the treatment experience and clinical outcomes of DMTs vary across different regions. Here, we present 5-year follow-up data on motor function, growth status and complications in a large Chinese paediatric 5q-SMA cohort treated with DMTs since December 2019, detailing their diverse clinical outcomes.

## Materials and methods

### Subjects

A total of 102 patients with 5q-SMA with DMTs in the department of paediatric endocrinology and genetic metabolism at the Guangzhou Women and Children’s Medical Center, China, were retrospectively analysed from 1 December 2019 to 31 December 2024. The diagnosis of 5q-SMA was confirmed by onset of clinical signs and symptoms, genetically analyses that met the guidelines published in 2007 as consensus statement for standard of care in SMA.^4^ Data obtained from medical records include clinical history, laboratory examinations, SMA genetic diagnosis and scores on motor functional outcome measures. Baseline information included, which involved *SMN1* mutations, *SMN2* copies, sex, age of symptom onset, age at diagnosis, age of first pharmacological therapies, body height and weight, respiratory support, scoliosis, feeding status and motor function, biochemical index and electrocardiogram.

This study involving all participants, materials and all clinical data has been performed in accordance with the Declaration of Helsinki and has been approved by the Ethics Committee of Guangzhou Women and Children’s Medical Center, Guangzhou Medical University (approval number: 20201221001). Written informed consent was obtained from the proband’s parents with the agreement to share the clinical and genetic information for research analysis.

### Treatment

Treatment with nusinersen was available from December 2019 for children with 5q-SMA. We applied lidocaine cream prior to lumbar intrathecal administration of nusinersen in patients of different, according to the recommended schedule with routine dose of nusinersen.^5^ Treatment with risdiplam was available from March 2023 for SMA patients aged 2 months and above. The dosing regimen of oral risdiplam is based on age and weight, and the medication is typically administered daily.^6^ In this 5-year observation period, there is no unified standard for nusinersen monotherapy and combined DMTs.

### Motor function evaluation

SMA patients underwent motor function assessments using the following tests: Children’s Hospital of Philadelphia Infant Test of Neuromuscular Disorder (CHOP INTEND)^7^ and Expanded Hammersmith Functional Motor Scale (HFMSE) test.^8,9^ All patients who received DMTs were assessed before pharmacological treatment. The evaluation scores of motor function before the last treatment were recorded. Clinically relevant improvements were regarded as an increase of no less than 3 points on the CHOP INTEND or HFMSE.^10^

### Physical examination and insulin-like growth factor-1 measurement

The height, weight and body mass index (BMI) were documented before each treatment. Height, weight and BMI were converted into height standard deviation scores (HtSDS), weight standard deviation scores (WtSDS) and BMI standard deviation scores (BMISDS) based on Chinese children and adolescent growth charts. Insulin-like growth factor-1 (IGF-1) was determined according to standard methods with the use of automated equipment. IGF-1 standard deviation scores (IGF-1SDS) were calculated based on the reference standard.^11^

### Follow-up and complication monition

This cohort of patients is under investigation every 4–6 months. Follow-up evaluations focused on motor function and the occurrence of potential complications, which included poor weight gain with growth failure, respiratory disease, scoliosis and fracture.^3^

### Statistical analysis

SPSS (version 26.0) software was used for statistical analysis. Data on baseline demographics and clinical characteristics were presented using descriptive statistics. To handle missing data, we employed the complete case analysis method, which involves analysing only the cases with complete data for all variables included in the analysis. The continuous normally distributed variables were expressed in terms of means and standard deviations (SD). The non-normally distributed variables were characterized by the median and range and compared using the Mann–Whitney *U* test. A statistical significance was defined as *P* <0 .05.

## Result

### Demographic and baseline clinical characteristics

One hundred and two SMA patients were enrolled in the study, including 24 patients with type 1 SMA, 56 with type 2 SMA and 22 with type 3 SMA. Demographic and baseline clinical characteristics of these patients are shown in Table 1. At the time of first injection, all patients were paediatric cases. Up to the final follow-up, two patients with type 3 SMA transitioned to adult hospital for continuous treatment. Genetic analysis identified 60.9% of SMA type 1 had 3 copies of *SMN2*, while 39.1% had 2 copies. 96.4% of SMA type 2 had 3 copies of *SMN2*. 57.2% of SMA type 3 patients had 3 copies of *SMN2*, and 42.8% had 4 copies of *SMN2*. All patients experienced symptom onset before treatment. The mean period from symptom onset to DMT initiation was 18, 36 and 61 months for SMA types 1–3, respectively.

**Table 1:** Clinical and genetic characteristics of SMA patients

	SMA type 1	SMA type 2	SMA type 3
Number of patients	24	56	22
Male sex, % (*n*)	83.3% (20)	48.21% (27)	59% (13)
Age at symptom onset (mean ± SD), months	3.8 ± 1.7	11.0 ± 4.1	23.0 ± 12.1
Age at diagnosis (mean ± SD), months	6.6 ± 3.5	16.4 ± 6.3	42.6 ± 33.5
Age at treatment initiation (mean ± SD), months	22.4 ± 26.2	47.4 ± 37.2	84.9 ± 56.3
Time interval between diagnosis and treatment (mean ± SD), months	16.7 ± 25.7	30.9 ± 38.6	42.3 ± 42.1
Treatment before 2023	21.0 ± 28.6	35.1 ± 40.0	42.3 ± 42.1
Treatment after 2023	4.0 ± 7.4	6.4 ± 10.8	
Respiratory support (*N*, %)	6 (25%)	1 (1.8%)	0
Feeding difficulties	2 (8.3%)	1 (1.8%)	0
Scoliosis	4 (16.7%)	12 (21.4%)	4 (18.2%)
SMN2 copies, *n* (%)	23	56	21
2	9 (39.1%)	0	0
3	14 (60.9%)	54 (96.4%)	12 (57.2%)
4	0	2 (3.6%)	9 (42.8%)

### Treatment and follow-up

**Figure 1:**
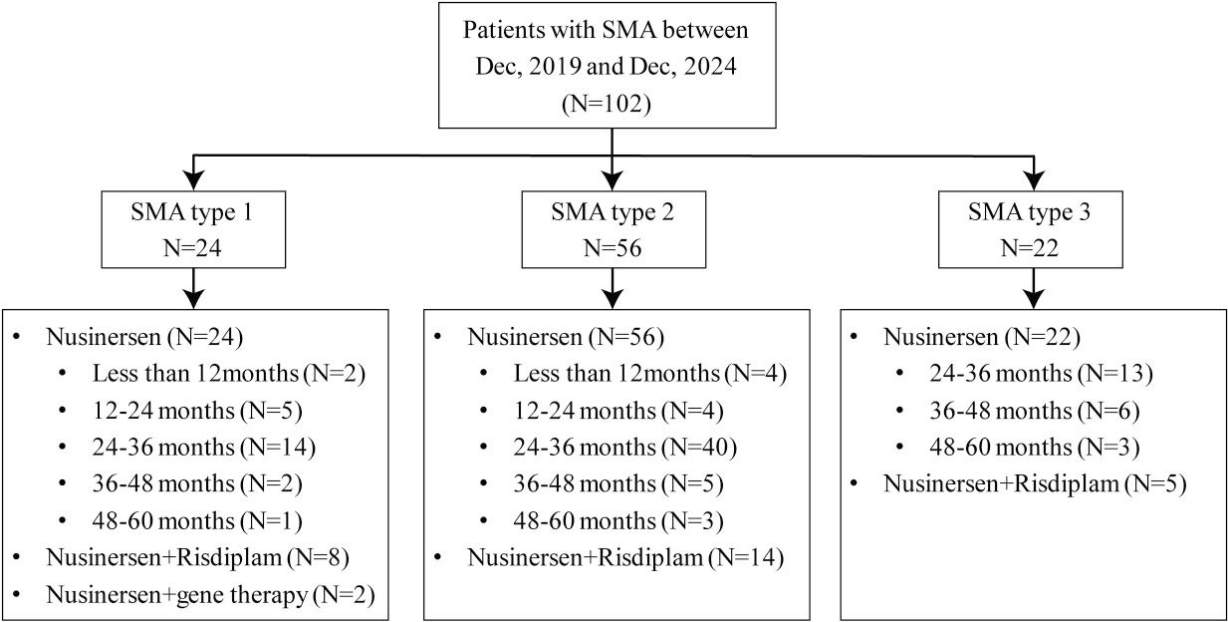
Overview of enrolled SMA patients.

All patients received nusinersen therapy after diagnosis. Of them, 6 SMA patients received nusinersen for less than 12 months, 9 received it for 12–24 months, 67 received it for 24–36 months, 13 for 36–48 months and 7 patients for 48–60 months. In addition, 30.3% (31/102) of them were treated with a combination of DMTs. Two with type 1 SMA underwent with onasemnogene abeparvovec overseas, and 30 patients started risdiplam treatment simultaneously since 2023 (described in Fig. 1).

During the 5-year follow-up, 5 patients with type 1, 13 with type 2 and 5 with SMA type 3 continued DMTs at their local hospitals. These patients are followed by telephone every 6 months. All remaining SMA patients are followed up every 4 months according to the treatment schedule.

### Motor function changes

For type 1 patients, the mean CHOP INTEND score at the start of treatment with disease-modifying drugs was 19.2 (±9.9) points, increasing to 34.4 (±15.5) points after a mean follow-up period of 30.4 (±11.4) months, which showed a significant statistical difference (*P* = 0.002). Five patients have achieved developmental milestones of sitting independently. For SMA type 2 and 3 patients, the mean HFMSE score at the start of treatment with disease-modifying drugs was 20.9 (±10.2) and 40.3 (±13.7) points, increasing to 28.0 (±13.1) and 49.8 (±12.5) points after a mean follow-up period of 33.5(±10.3) and 37.7(±9.7) months, which both showed significant statistical difference (*P* = 0.007; *P* = 0.008), respectively. Four patients with type 2 SMA who had never been able to walk achieved independent ambulation after DMTs.

### Body metrics and IGF-1 changes

The body metrics and IGF-1 changes were presented in Table 2. After treatment, type 2 and 3 SMA patients exhibited differing extents of improvement in weight and IGF-1 levels. The mean WtSDS for patients with type2 and 3 SMA at the start of treatment with DMTs were −1.63 and −1.03 SD, increasing to −1.55 and −0.97 SD, respectively, which were showed no statistical significance. (*P* = 0.811 and *P* = 0.888). The levels of IGF-1 for SMA type 2 were significantly increased (*P* < 0.05). However, no significant effect in IGF-1 level was observed in patients with type 1 or type 3 SMA (*P* = 0.30, *P* = 0.24). The declines in HtSDS compared with baseline were seen in type 1 and 2 patients.

**Table 2:** Body metrics and IGF-1 level of SMA patients before and after therapy

	SMA type 1 (*n* = 24)		SMA type 2 (*n* = 56)		SMA type 3 (*n* = 22)	
	Before	After	Before	After	Before	After
Motor function, mean (SD)	19.2 ± 9.9^a^	34.4 ± 15.5^a^	20.9 ± 10.2^b^	40.3 ± 13.7^b^	28.0 ± 13.1^b^	49.8 ± 12.5^b^
Height, cm, mean (SD) (min, max)	79.9 ± 17.9 (55 130)	96.9 ± 17.2 (72 140)	93.7 ± 15.2 (69 135)	105.3 ± 16.7 (76 150)	115.5 ± 24.7 (85 171)	127.8 ± 22.4 (98.5,175.0)
HtSDS, mean (SD) (min, max)	−0.75 ± 2.79 (−7.80, 2.50)	−1.62 ± 1.51 (−5.4, 1.5)	−1.01 ± 1.33 (−3.90, 2.30)	−1.13 ± 1.31 (−5.7, 2.8)	−1.39 ± 1.73 (−6.50, 1.10)	−1.29 ± 1.14 (−3.10, 1.50)
Weight, kg, mean (SD) (min, max)	9.5 ± 4.0 (4.5, 24.5)	13.4 ± 4.6 (8.5 ± 28)	13.4 ± 4.7 (6.3, 26.0)	16.9 ± 6.2 (8.2, 34.0)	23.7 ± 14.0 (9.9, 58.6)	29.41 ± 16.47 (11.8, 74.0)
WtSDS, mean (SD) (min, max)	−1.58 ± 3.02 (−6.70, 2.90)	−2.41 ± 1.88 (−5.70, 1.10)	−1.63 ± 1.85 (−7.00, 2.20)	−1.55 ± 1.74 (−6.60, 1.90)	−1.03 ± 1.25 (−2.80, 1.30)	−0.97 ± 1.53 (−3.70, 1.60)
BMI, kg/m^2^, mean (SD) (min, max)	14.7 ± 2.7 (9.6, 19.8)	13.9 ± 2.0 (9.5, 17.4)	14.5 ± 2.3 (7.1, 19.0)	14.6 ± 2.5 (9.5, 22.8)	16.2 ± 3.2 (12.1, 25.0)	16.6 ± 3.8 (12.0, 24.2)
BMI SDS, mean (SD) (min, max)	−1.50 ± 2.50 (−7.10, 1.80)	−1.8 ± 2.4 (−8.20, 1.30)	−1.31 ± 2.42 (−10.80, 2.10)	−1.09 ± 2.07 (−7.00, 2.60)	−0.36 ± 1.39 (−3.00, 1.90)	−0.45 ± 1.62 (−3.70, 1.80)
IGF-1, μmol/L, mean (SD) (min, max)	41.5 ± 17.7 (21.6, 68.9)	80.4 ± 39.1 (33.3, 146.0)	108.8 ± 60.1 (29.3, 335.0)	135.4 ± 49.3 (50.8, 239.0)	159.9 ± 84.8 (45.4, 298.0)	188.0 ± 92.6 (63.6)
IGF-1SDS, mean (SD) (min, max)	−1.60 ± 0.95 (−2.77, 0.85)	−0.87 ± 0.91 (−1.79, 0.72)	−0.58 ± 1.13 (−4.35, 1.79)	0.35 ± 1.24 (−2.74, 3.14)	−1.13 ± 1.18 (−3.59, 1.80)	−0.45 ± 1.29 (−1.86, 3.56)

^a^SMA type 1 were used CHOP INTEND to assess motor function.

^b^SMA type 2 and 3 were used HFMSE to assess motor function.

### Survival and complication

Through the study period, two patients with SMA type 2 and one SMA type 1 died. 62.7% (64/102) of all group of SMA patients exhibited either one or two complications, while 13.7% (14/102) developed skeletal, gastrointestinal and respiratory complications, presenting with scoliosis, malnutrition and pulmonary infection. In type 1 group, 10 patients suffered pneumonia, among whom six patients required assisted respiratory therapy. Fifteen patients developed malnutrition, nine of whom progressed to severe malnutrition. Seventy-five per cent (18/24) of the patients presented scoliosis. One patient sustained one fracture and ultimately succumbed to severe pneumonia. In type 2 and 3 SMA group, 60% (34/56) and 54.5% (12/22) of patients had scoliosis, 28.5% (16/56) and 9.1% (2/22) experienced pneumonia and 37.5% (21/56) and 27.2% (6/22) were malnourished. Six patients with type 2 needed assisted respiratory therapy.

### Association between time to treatment initiation and diverse clinical outcomes

The mean time from symptom onset to DMT initiation in patients with type 1–3 SMA was 16.7, 30.9 and 42.3 months, respectively. Table 3 showed type 1 patients who received DMTs within 6 months of symptom onset (*n* = 13) demonstrated a mean improvement of 25 points in the CHOP INTEND score. Conversely, patients who received nusinersen treatment 6 months after symptom onset (*n* = 11) exhibited a mean improvement of 5 points in the CHOP INTEND score. In patients with type 2 and type 3 SMA, a shorter time to treatment initiation is associated with greater magnitude of motor function improvement (a higher HFMSE score). Meanwhile, prolongation of the time from symptom onset to DMT initiation increased the occurrence of complications, including scoliosis, pneumonia, malnutrition and mortality.

**Table 3:** Association between time to treatment initiation (TTI) and clinical outcomes

TTI	Patient	Motor function changes, mean (SD)	Scoliosis, (*N*, %)	Pneumonia, (*N*, %)	Assisted respiratory therapy (*N*, %)	Malnutrition (WtSDS from −2SD to −3SD) (*N*, %)	Severe malnutrition (WtSDS < −3SD) (*N*, %)	Fracture (*N*, %)	Death (*N*, %)
Type 1 SMA									
TTI ≤ 6 months	13	25.53 ± 10.78^a^	8 (61.5%)	3 (23.1%)	1 (33.3%)	7 (53.8%)	2 (28.5%)	0	0
TTI > 6 months	11	5.36 ± 7.35^a^	10 (90.9%)	7 (63.6%)	5 (71.4%)	8 (72.7%)	7 (87.5%)	1 (9.1%)	1 (9.1%)
Type 2 SMA									
TTI ≤ 18 months	22	16.31 ± 9.37^b^	12 (54.5%)	3 (13.6%)	2 (66.7%)	6 (27.3%)	2 (33.3%)	0	0
TTI > 18 months	34	8.27 ± 5.50^b^	22 (64.7%)	13 (38.2%)	4 (30.8%)	12 (35.2%)	4 (33.3%)	0	2 (5.9%)
Type 3 SMA									
TTI ≤ 36 months	11	11.90 ± 6.67^c^	8 (8/10, 80%)^d^	0	0	2 (18.2%)	2 (100%)	0	0
TTI > 36 months	11	6.82 ± 5.07^c^	4 (4/7, 57.1%)^e^	1 (9.1%)	0	4 (36.3%)	1 (25%)	0	0

^a^Motor scores differed significantly between patients with a TTI ≤ 6 months and those with a TTI > 6 months (*P* = 0.001).

^b^Motor scores differed significantly between patients with a TTI ≤ 18 months and those with a TTI > 18 months (*P* = 0.001).

^c^Motor scores differed significantly between patients with a TTI ≤ 36 months and those with a TTI > 36 months (*P* = 0.001).

^d^Spinal radiographs were repeated in 10 of the 11 patients.

^e^Spinal radiographs were repeated in 7 of the 11 patients.

## Discussion

Nusinersen and risdiplam have been confirmed to be effective and safe in international cohort studies.^12,13^ The efficacy and safety of nusinersen in a 2-year follow-up Chinese paediatric cohort were subsequently reported in 2024 through a multi-centre registry study, which did not include data from our centre.^14^

This study included 102 Chinese SMA paediatrics and retrospective examined motor function, growth, complication and mortality over a 5-year follow-up period. Given the comprehensive nature of the data collected and the relatively large sample size over an extended follow-up period, the findings from this study may offer valuable insights into the long-term outcomes of SMA in paediatric populations. Throughout the DMT period, 76.4% of patients developed at least one complication. Three patients (2.9%) died from their complications. Overall survival was 97.1%, aligning with data from published international cohorts.^15^ Despite the critical importance of prompt treatment following diagnosis, treatment initiation in this cohort was consistently delayed, especially among patients diagnosed before 2023. The average time from the symptom onset to diagnosis was 3 months for type 1, 5 months for type 2 and 19 months for type 3. The mean time from diagnosis to first dose of DMTs was 16 months for type 1, 30 months for type 2 and 42 months for type 3. Based on current evidence, time to treatment initiation was a single strongest modifiable predictor of therapeutic outcome in SMA.^16^ Our data showed clinically significant changes in motor function scores for type 1 SMA patients who received DMTs within versus after 6 months of symptom onset. Similar pattern was observed in type 2 patients. In addition, patients who received delayed treatment had a higher incidence of malnutrition, pneumonia and scoliosis. 27.4% (28/102) of patients suffered pneumonia during follow-up. Twelve patients (42.8%) suffered serious pneumonia and needed for ventilator support, including six type 1 or 2 patients, respectively. Two patients die of severe pneumonia. Chen *et al.* published a 2-year single-centre experience and highlighted that children with SMA type 1 treated with nusinersen continued to develop respiratory comorbidities.^17^ In a meta-analysis of 15 eligible studies, Zhong *et al.* reported that pneumonia affected up to 26.6% of patients, which was not only an additional morbidity, but also a potential lethal complication.^18^

Our study summarized the growth status of patients with SMA before and after treatment, including height and weight based anthropometric indices and IGF-1 levels. First, we assessed body weight. Patients with type 1 disease exhibited weight loss, whereas those with types 2 and 3 showed modest, but non-significant, weight gains. Current evidence indicates that changes in body weight among patients with SMA during treatment are associated with multiple factors. Type 1 SMA progress to malnutrition as a result of inadequate energy and protein intake, while type 2 and type 3 SMA are more likely to become overweight or obese, especially those with type 3 SMA, who have the highest risk of overweight or obesity.^19^

Second, the mean height of patients with type 1 and 2 SMA decreased after treatment compared to baseline in this study, which is attributable to the disease’s intrinsic capacity to independently constrain linear growth. Moreover, the incidence of scoliosis increased during treatment. Among type 1 patients, scoliosis prevalence rose from 16.6% pre-treatment to 75% post-treatment and among type 2 patients, from 21.4 to 60%. This progressive spinal deformity is a key contributor to the observed loss of height. What is more, IGF-1 plays a crucial role in linear growth and development in childhood^20,21^; therefore, we focused on and monitored its changes during DMTs. In this study, although post treatment IGF-1 levels increased, the rise did not reach statistical significance; consequently, no linear growth spurt was observed in type 1 patients.

Regarding motor function, most patients showed a positive response, with abilities either improving or remaining stable, consistent with earlier reports.^22-24^

In summary, this study reinforces on long-term follow-up clinical outcomes of approved DMTs for SMA in China. Nusinersen and risdiplam are effective on motor function. Nevertheless, relying solely on DMTs offer limited leverage over the persistent impairment of growth and remains difficult to prevent the onset of skeletal, respiratory or gastrointestinal complications. Early diagnosis followed by prompt initiation of DMTs, especially presymptomatic treatment, is critical. However, our research currently lacks data on presymptomatic treatment. Therefore, we emphasize the future investigations to focus on this area in order to better understand the potential long-term effects of presymptomatic treatments.

## Data Availability

Data sharing is not applicable to this article as no new data were created or analysed in this study.
